# Exploring the temporal dynamics of circadian rhythm-aligned VR intervention in mitigating fear of cancer recurrence: mediating roles of illness uncertainty and hope in gynecological cancer patients

**DOI:** 10.3389/fpsyg.2026.1797203

**Published:** 2026-04-13

**Authors:** Bo Wang, Hongmei Xu, Lanlan Zhang, Junwei Wang, Xiangqin Ji, Shunlian Luan, Shuhong Shao

**Affiliations:** 1 Binzhou Medical University-Yantai Campus, Yantai, China; 2 Binzhou Medical University Hospital, Binzhou, China

**Keywords:** circadian rhythm, fear of cancer recurrence, gynecological cancer, hope, illness uncertainty, oncological care, virtual reality

## Abstract

**Background:**

Fear of cancer recurrence (FCR) is a prevalent and clinically significant psychological burden that adversely affects the quality of life and treatment adherence among gynecological cancer survivors. Virtual reality (VR), an emerging therapeutic modality, has demonstrated potential applications in oncology-related psychological interventions. This study investigates the efficacy of a chronotype-based timed VR intervention in alleviating FCR and explores the mediating mechanism of illness uncertainty (IU) and hope.

**Methods:**

A total of 63 patients were randomly allocated to either the VR intervention group (*n* = 31) or the control group (*n* = 32). Both groups received standard medical care. The intervention group completed a 4-week VR intervention (5 sessions per week, 20 min per session) scheduled according to individual chronotypes, which were assessed via the Morningness-Eveningness Questionnaire. Outcome measures, including FCR, IU, and hope, were collected at baseline, 1 week (T1), 1 month (T2), and 3 months (T3) after the intervention. Data were analyzed using ANCOVA and PROCESS macro for mediation effects.

**Results:**

Baseline characteristics were well balanced (all *p* > 0.05). Compared with controls, the VR group had significantly lower FCR scores at T1 (mean difference = 4.11), T2 (6.03), and T3 (5.86) (all *p* < 0.001), with large effect sizes (partial *η^2^* = 0.535–0.617). Mediation analyses revealed dynamic mechanisms: T1 (parallel/chain mediation by IU and hope, accounting for 74.7% of total effect); T2 (only IU mediated, 50.3%); T3 (direct effect dominant, 82.0%).

**Conclusion:**

A chronotype-based timed virtual reality intervention effectively reduces fear of cancer recurrence in gynecological cancer patients and acts through mediating pathways by reducing illness uncertainty and enhancing the level of hope. These findings provide empirical support for integrating chronotype-aligned virtual reality intervention into clinical practice and underscore the importance of considering temporal dynamics in psychological intervention design.

## 1 Introduction

Globally, the burden of cancer continues to rise steadily, driven primarily by population growth and aging (Bray et al., 2024; Ju et al., 2023). Simultaneously, advances in early detection and therapeutic interventions have significantly improved survival rates, resulting in a growing population of cancer survivors who face enduring psychosocial challenges (Egger-Heidrich et al., 2025). Among these contexts, fear of cancer recurrence (FCR), the most prevalent psychological concern, impacts approximately 59% of patients (Luigjes-Huizer et al., 2022). Consequently, FCR has emerged as a critical target in cancer rehabilitation and survivorship care.

Gynecological cancers are major diseases threatening women’s health, and they show particularly high FCR after patients undergo comprehensive treatments such as surgery, chemotherapy, and radiotherapy. This is linked to the unique disease and treatment-related factors of gynecological cancers. For instance, some ovarian cancer subtypes have a recurrence rate of up to 90% (Ebrahimi et al., 2024). Pelvic radiotherapy and other treatment modalities may lead to long-term side effects (Pizzo et al., 2024). Moreover, the treatments can also have an impact on fertility (e.g., when considering fertility preservation plans such as ovarian tissue transplantation) (Erden et al., 2024). These factors intensify the psychological burden of gynecological cancer patients, leading to FCR issues with strong persistence and high intensity. This excessive worry about cancer recurrence is not only closely linked to emotional disorders such as anxiety and depression but also significantly influences patients’ quality of life, social function recovery, and long-term treatment compliance (Gross et al., 2024). Nevertheless, current specialized research on FCR in gynecological cancer patients remains relatively limited, especially in terms of mechanism exploration and intervention development. Most current studies primarily rely on the experience from other cancer types, but fail to fully consider the unique disease characteristics and the particularity of the patient group. Thus, there is a need for clinical attention and effective intervention measures (Ng et al., 2025; Sinha et al., 2023).

From a theoretical standpoint, illness uncertainty (IU) and hope level could be two crucial factors in comprehending the maintenance mechanism of FCR. Within the framework of the Common Sense Model of Self-Regulation, IU reflects patients’ indistinct cognition of the prognosis of their illness (Hagger and Orbell, 2022), which may lead to a negative hypervigilance-avoidance cycle. In contrast, hope level represents a positive psychological resource, which can mitigate the impact of threat perception by enhancing an individual’s goal setting capacity and pathway thinking (Xiong et al., 2025). These two factors offer important theoretical perspectives for understanding the occurrence and maintenance of FCR, respectively, from the dimensions of cognitive assessment and emotional resources.

Traditional interventions, such as mindfulness and cognitive behavioral therapy, have demonstrated a certain degree of efficacy in dealing with the FCR. However, these methods face implementation challenges, especially in simulating anxiety situations and providing personalized care, which has limited their effects (Carlson et al., 2023). In contrast, virtual reality (VR) technology, with its immersive and controllable environments, presents a promising solution. Research indicates that it can alleviate anxiety through exposure and distraction therapies and foster hope via positive emotional experiences such as deep immersion and narrative engagement (Chan et al., 2024). Furthermore, recent advancements in chronobiology have offered a novel direction for optimizing psychological interventions. Evidence suggests that individuals’ emotional regulation capacities and cognitive resources fluctuate significantly in accordance with circadian rhythms. Tailoring the timing of interventions to one’s biological rhythm characteristics may substantially enhance the efficacy (Steardo et al., 2025). Nevertheless, in the context of FCR interventions for gynecological cancer patients, studies integrating circadian personalization with VR remain scarce.

Based on the above research background, this study conducted a randomized controlled design to examine the intervention effect and mechanism of personalized VR intervention based on circadian rhythm on FCR in gynecological cancer patients. The following research hypotheses are proposed:

*Hypothesis 1*: Compared with standard medical care, the VR intervention will significantly mitigate FCR symptoms in gynecological cancer patients across follow-up periods.

*Hypothesis 2*: IU and hope levels will, respectively, serve as mediating factors in the relationship between the VR intervention and FCR reduction.

*Hypothesis 3*: The mediating mechanisms of IU and hope will undergo dynamic changes across the 1-week (T1), 1-month (T2), and 3-month (T3) follow-up stages.

These research findings will provide empirical evidence for the development of precise psychological intervention programs, and promoted the innovative development of psychological rehabilitation services for gynecological cancer patients.

## 2 Methods

### 2.1 Study setting and sampling

This study was conducted from March 2025 to October 2025 at the Department of Gynecologic Oncology and Pelvic Surgery of Binzhou Medical University Hospital. The participants were recruited through convenience sampling from patients receiving inpatient care and were diagnosed with gynecologic cancers.

To determine the required sample size, we first estimated an effect size of 0.20 based on existing literature, then used G*Power V.3.1.9.2 software for calculation. Assuming an error probability (*α*) of 0.05 and statistical power (1−β) of 0.90, a repeated-measures ANOVA (two groups, three time points) required a minimum of 56 participants to detect this effect size (Hayes, 2017). Accounting for a 10% dropout rate (consistent with prior research), we set an initial recruitment target of 64 participants (32 per group).

### 2.2 Inclusion and exclusion criteria

Inclusion criteria: (1) Aged 35–70 years, with pathological diagnosis of cervical, ovarian, or endometrial cancer (ICD-10 cis scale was developed by Horne JA and Ostodes: C53–C55), and ≥3 months since initial diagnosis; (2) Clear consciousness, sufficient communication/comprehension abilities, and capacity to independently complete questionnaire assessments; (3) Voluntarily provided written informed consent and committed to completing all follow-up procedures; (4) Completed initial treatment (surgery, chemotherapy, or radiotherapy) for ≥1 month, with clinically stable disease (no evidence of active tumor progression).

Exclusion Criteria: (1) Comorbid severe psychiatric or cognitive disorders; (2) Significant visual/auditory impairments, vertigo, or other contraindications to virtual reality (VR) intervention; (3) Currently pregnant or lactating; (4) Participation in other psychological interventions or oncological rehabilitation-related clinical trials within the past 3 months.

Sixty-four participants were initially enrolled. One participant withdrew prior to intervention commencement, resulting in 63 participants who were randomly assigned. Demographic, clinical, and baseline psychological characteristics were collected to assess between-group comparability.

### 2.3 Randomization and blinding

Following baseline assessment, participants were randomly assigned to either the VR intervention group or the control group using a computer-generated block randomization sequence with block size of 4, prepared by an independent statistician not involved in participant recruitment or intervention delivery. Allocation concealment was achieved through sequentially numbered, opaque, sealed envelopes, which were managed by an independent research assistant. Group assignment was revealed only after the envelope was opened at the time of enrollment. Outcome assessors and data analysts were blinded to group allocation throughout the study. A blinded verification was conducted at study completion to confirm the effectiveness of blinding (guessing accuracy rate: 47%, *p* = 0.776).

### 2.4 Study interventions

This study adopted a parallel-group controlled design. Both groups received routine cancer care, which encompassed health education associated with cancer treatment, guidance on the management of adverse reactions, routine psychological support, and regular follow-up. On this foundation, the intervention group received an additional four-week personalized VR psychological intervention using the hospital’s standardized VR psychotherapy device (PICO G2 4K, equipped with integrated biofeedback sensors for real-time heart rate variability monitoring).

The intervention program was refined by a multidisciplinary expert group (comprising 1 chief physician of oncology, 2 associate chief physicians of oncology, 5 specialized nurses of oncology, 2 chief physicians of psychology, and 1 attending psychiatrist) to ensure scientific rigor, clinical suitability, and safety. The intervention targeted the hypothesized mechanisms underlying FCR reduction through three evidence-based modules: (1) cognitive restructuring to address illness uncertainty, (2) emotion regulation training to mitigate FCR-associated distress, and (3) positive future-oriented visualization to foster hope. The detailed content, session structure, and progression plan are presented in Supplementary material, which specifies the theoretical foundation, core activities, and intended mechanistic outcomes for each module.

**Figure 1:**
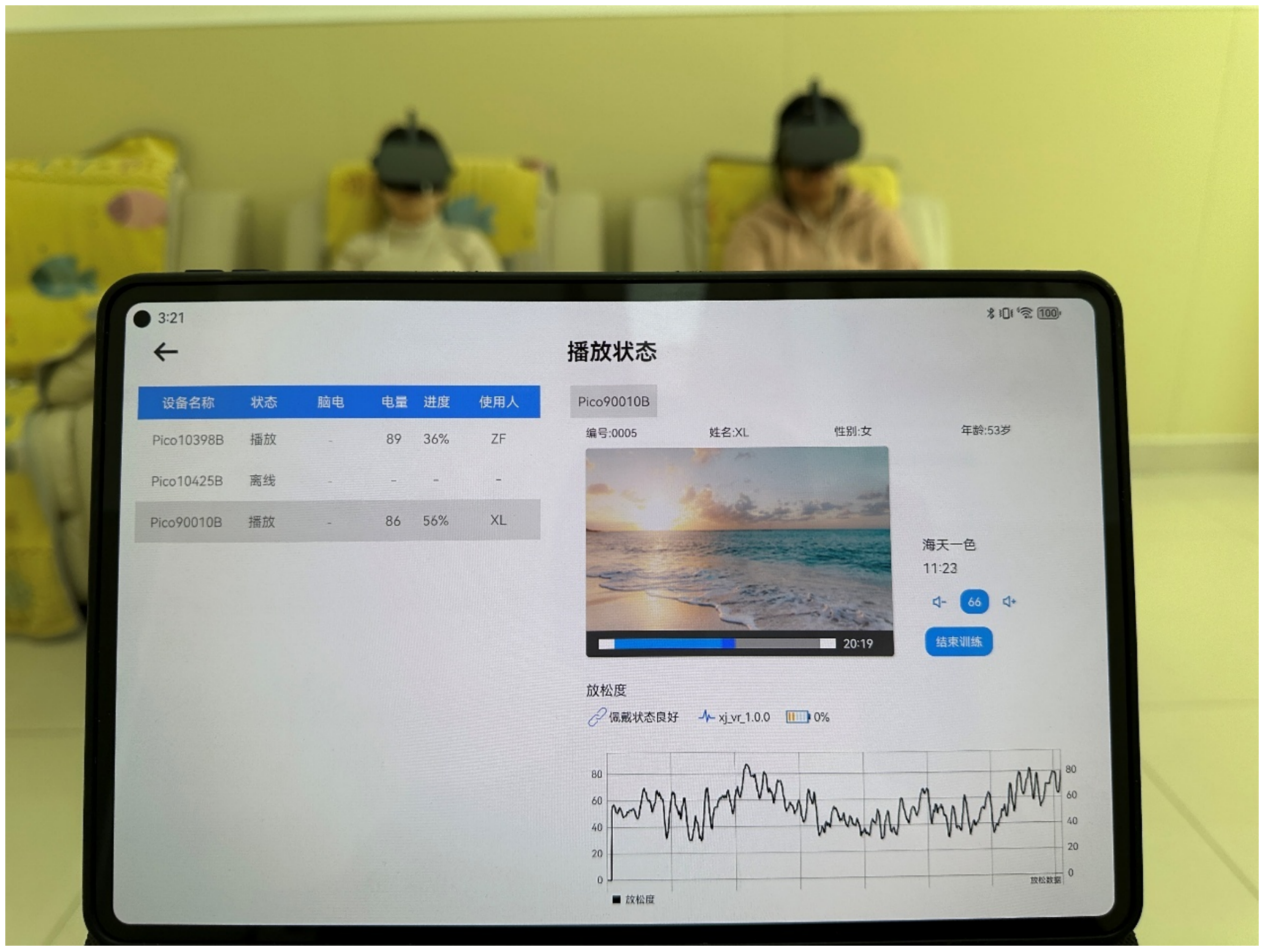
Implementation process of the circadian rhythm-personalized VR intervention.

**Figure 2:**
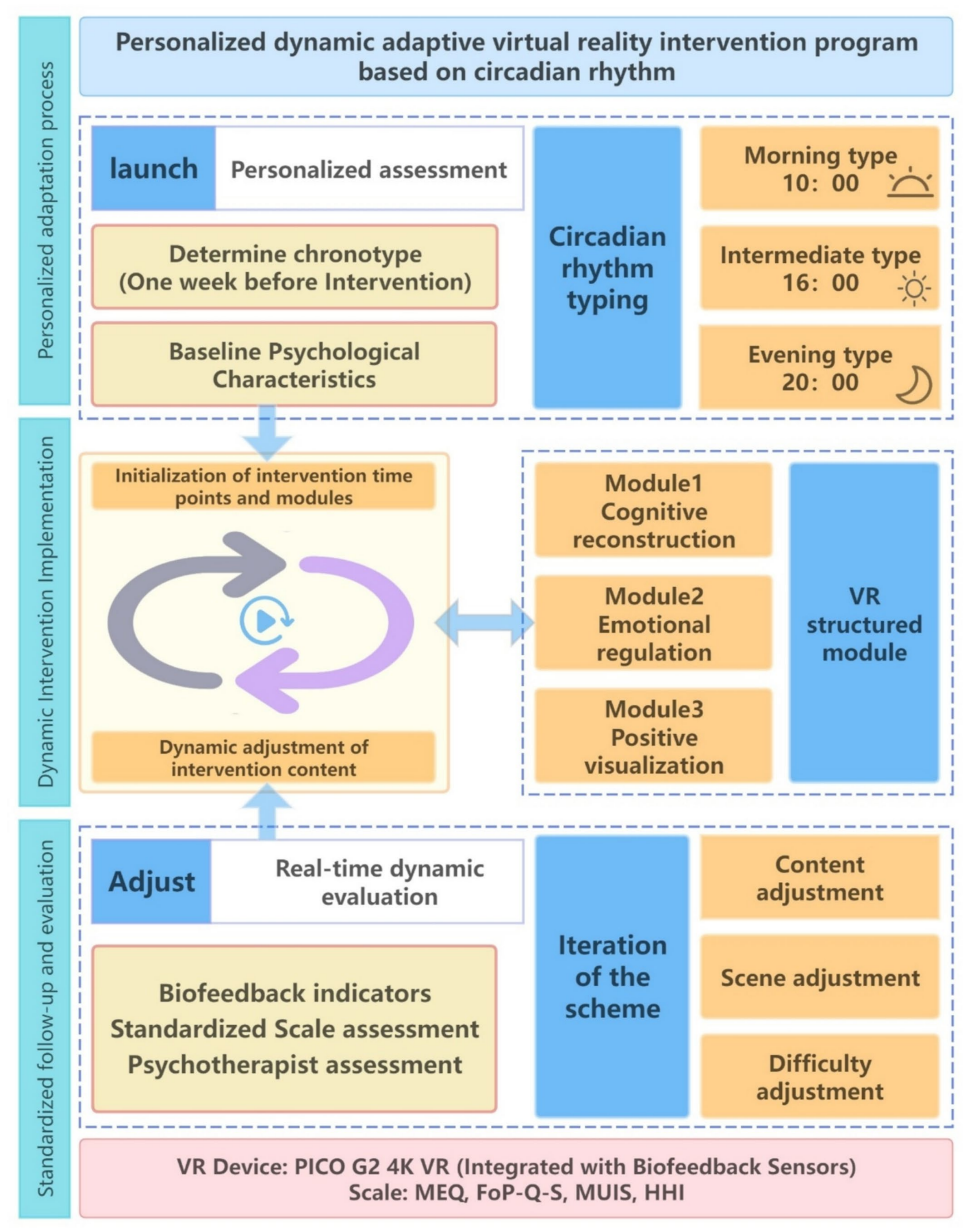
Flowchart of the circadian rhythm and biofeedback-based personalized VR psychological intervention study design. MEQ, Morningness-Eveningness Questionnaire; FoP-Q-SF, Fear of Progression Questionnaire–Short Form; MUIS, Mishel Uncertainty in Illness Scale; HHI, Herth Hope Index; HRV, Heart rate variability.

One week before the intervention began, the Morningness-Eveningness Questionnaire (MEQ) was used to assess patients’ chronotypes, and participants were classified into morning, intermediate, or evening types. Personalized intervention time slots were subsequently assigned: 10:00 for morning types (corresponding to their peak period for cognitive and emotional regulation), 16:00 for intermediate types (to minimize discomfort during the day-night transition), and 20:00 for evening types (to align with their nocturnal preference and enhance adherence). Based on HRV metrics from the biofeedback sensors, the system made micro-adjustments to session pacing and guidance narration to optimize patient engagement and relaxation responses. The intervention was conducted 5 sessions per week for 4 weeks (20 sessions total), with each session lasting 20 min (see Figure 1). Figure 2 depicts its complete intervention process design.

Each 20-min session followed a standardized structure: 3-min introduction, 14-min core activity, and 3-min summary reflection. Intervention fidelity was maintained through weekly supervision meetings with the principal investigator, random session observations by licensed psychotherapists (10% of sessions), and completion of fidelity checklists after each session. Therapists were responsible for pre-session device setup and patient orientation, real-time monitoring of patient responses during VR exposure, and post-session debriefing to reinforce learning points.

### 2.5 Measures

The Morningness-Eveningness Questionnaire (MEQ) was employed to assess the patients’ circadian rhythm types. This scale was developed by Horne JA and Ostberg O in 1976, and was translated into Chinese by Zhang Bin (Zhang et al., 2006). Subsequently, it has been extensively utilized in clinical research (Gonzalez and Valente, 2023). Fear of cancer recurrence was assessed using the 12-item Fear of Progression Questionnaire-Short Form (FoP-Q-SF) (Mehnert et al., 2006; Wu et al., 2015), scored on a 5-point Likert scale (1 = strongly disagree, 5 = strongly agree). Total scores range from 12 to 60, with higher scores indicating greater fear. Cronbach’s *α* ranged from 0.802 to 0.823 for subscales and was 0.878 for the total scale. IU was measured using the Chinese version of the Mishel Uncertainty in Illness Scale (MUIS), translated by Hsu Shu-lie (Sheu and Hwang, 1996), which contains 25 items rated on a 5-point scale (1 = strongly disagree, 5 = strongly agree). Scores range from 25 to 125, with higher values indicating greater uncertainty. Subscale *α* values ranged from 0.866 to 0.898, and overall internal consistency was 0.942. Hope level was assessed using the Herth Hope Index (HHI) (Wang, 2010), a 12-item scale with a 4-point response format (1 = strongly disagree, 4 = strongly agree). Total scores range from 12 to 48, with higher scores reflecting greater hope. The HHI showed a Cronbach’s *α* of 0.902 in this study.

### 2.6 Data collection

Outcome measures were collected at four time points: baseline (before intervention), 1 week post-intervention (T1), 1 month post-intervention (T2), and 3 months post-intervention (T3). All assessments were administered by trained oncology nurses using standardized protocols. Detailed randomization and blinding procedures are described in Section 2.2.

Data collectors completed a 16-h standardized training program before study commencement to ensure consistency in administration. All questionnaires were administered in paper format, with double-entry verification for data accuracy.

### 2.7 Statistical analysis

Data analysis was conducted using SPSS 27.0, with statistical significance set at *α* = 0.05 (two-tailed). Summary statistics were generated for demographic, clinical, and key variables at each time point. The comparability of baseline groups was assessed using independent samples *t*-tests or Mann–Whitney U tests for continuous variables and *χ^2^* or Fisher’s exact tests for categorical variables, as appropriate. ANCOVA was used to evaluate the VR intervention’s effect on FCR at each time point, adjusting for baseline values and potential confounders. Corrections were applied when variance homogeneity assumptions were violated. Mediation effects of IU and hope were analyzed using PROCESS macro (version 4.2) models 4 and 6, with 95% confidence intervals based on 10,000 bootstrap resamples.

### 2.8 Ethical considerations

This study was approved by the Ethics Committee of Binzhou Medical University (Approval Number: 2025-L062) and adhered to the Declaration of Helsinki and International Psycho-Oncology Society ethical guidelines for psychological oncology research. The data were anonymized with unique codes and stored on a secure hospital server with restricted access. All virtual reality sessions were conducted under the supervision of a licensed psychotherapist, who would monitor and record any adverse reactions in real time. To ensure the fairness of the research, after the end of the period, the participants in the control group received standardized VR-based psychological intervention.

## 3 Results

### 3.1 Characteristics of the sample

**Figure 3:**
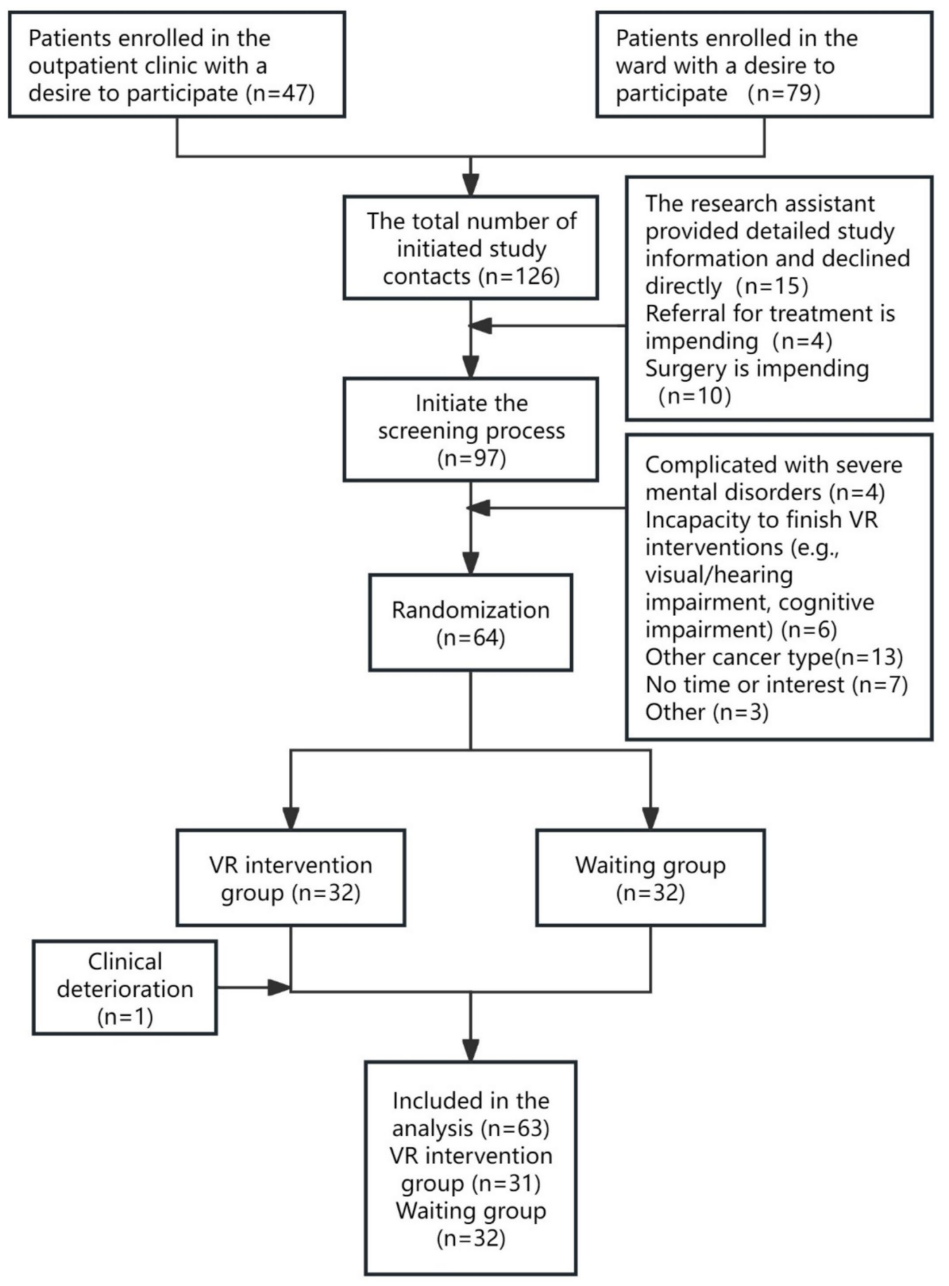
Flowchart of participant enrollment, screening, and retention.

Data were collected from March 2025 to October 2025. A total of 64 patients diagnosed with gynecological tumors were enrolled in the study. One participant experienced clinical deterioration on the sixth day of the VR intervention and subsequently withdrew. Ultimately, 63 participants completed the study and were included in the data analysis, with 31 assigned to the VR intervention group and 32 to the control group. Complete baseline data were obtained for all participants, with no missing data or post-enrollment withdrawals. The recruitment and retention of the participants are shown in Figure 3.

Baseline characteristics, including demographic variables, clinical treatment backgrounds, and core psychological outcomes, were similar between groups in terms of key parameters (Table 1). Demographically, these two groups were comparable in age (53.29 ± 7.45 vs. 52.81 ± 7.44, *p* = 0.800) and marital status. Clinically, no significant differences were observed in tumor type or disease stage. Psychologically, baseline scores for FCR, hope, and IU were similar between groups.

**Table 1:** Baseline characteristics of participants in both groups.

Variable	VR group (*n* = 31)	Control group (*n* = 32)	*p*
Demographics			
Age (years, Mean ± SD)	53.29 ± 7.45	52.81 ± 7.44	0.800
Marital status, *n* (%)			0.613*
Married	29 (93.5)	31 (96.9)	
Divorced/Widowed	2 (6.5)	1 (3.1)	
Education level, *n* (%)			0.618
College or above	6 (19.4)	4 (12.5)	
High school	12 (38.7)	11 (34.4)	
Junior high or below	13 (41.9)	17 (53.1)	
Medical payment method, *n* (%)			0.129
Partial self-payment	22 (71.0)	28 (87.5)	
Full reimbursement	9 (29.0)	4 (12.5)	
Clinical and treatment characteristics			
Months Since Diagnosis	18.87 ± 12.29	15.03 ± 8.11	0.147
Cancer Type, *n* (%)			0.759
Cervical cancer	8 (25.8)	11 (34.4)	
Ovarian cancer	13 (41.9)	12 (37.5)	
Endometrial cancer	10 (32.3)	9 (28.1)	
Disease stage, *n* (%)			0.131
Early (I + II)	20 (64.5)	14 (43.8)	
Advanced (III + IV)	11 (35.5)	18 (56.2)	
Cancer recurrence, *n* (%)			0.509*
No	25 (80.6)	28 (87.5)	
Yes	6 (19.4)	4 (12.5)	
Chemotherapy, *n* (%)			0.732*
No	4 (12.9)	6 (18.8)	
Yes	27 (87.1)	26 (81.2)	
Radiotherapy, *n* (%)			0.446
No	11 (35.5)	15 (46.9)	
Yes	20 (64.5)	17 (53.1)	
Baseline outcome measures			
Fear of cancer recurrence	28.48 ± 8.465	28.41 ± 9.648	0.973
Hope level	30.68 ± 7.507	30.50 ± 6.735	0.922
Illness uncertainty	58.45 ± 16.172	58.66 ± 17.232	0.961

Data are shown as mean ± SD for continuous variables and n (%) for categorical variables. Statistical tests: independent t-test (continuous); Pearson χ^2^ test (categorical, expected frequencies ≥5); Fisher’s exact test (*, expected frequencies <5).; Cohen’s d < 0.5 indicated no clinically significant difference.

Independent samples t-tests and Pearson χ^2^/Fisher’s exact tests were used to compare baseline characteristics. No significant between-group differences were found for any variable (all *p* > 0.05). All Cohen’s d values were less than 0.5, indicating negligible effect sizes and no clinically meaningful baseline disparities. This balanced profile provides a solid foundation for evaluating the subsequent intervention effects.

Intervention adherence was monitored throughout the 4-week intervention period. All participants in the VR group (31/31, 100%) completed the full intervention protocol. Session duration compliance rate was 93.7% (defined as≥18 min per session). Non-compliance was primarily attributed to acute chemotherapy-related adverse reactions (*n* = 17), temporary equipment malfunction (*n* = 6), and interruption by urgent clinical nursing procedures (*n* = 16). Notably, no non-compliance instances were due to conflicts between the assigned time slots and patients’ medical schedules or personal routines.

### 3.2 Main effect of VR intervention on FCR

**Figure 4:**
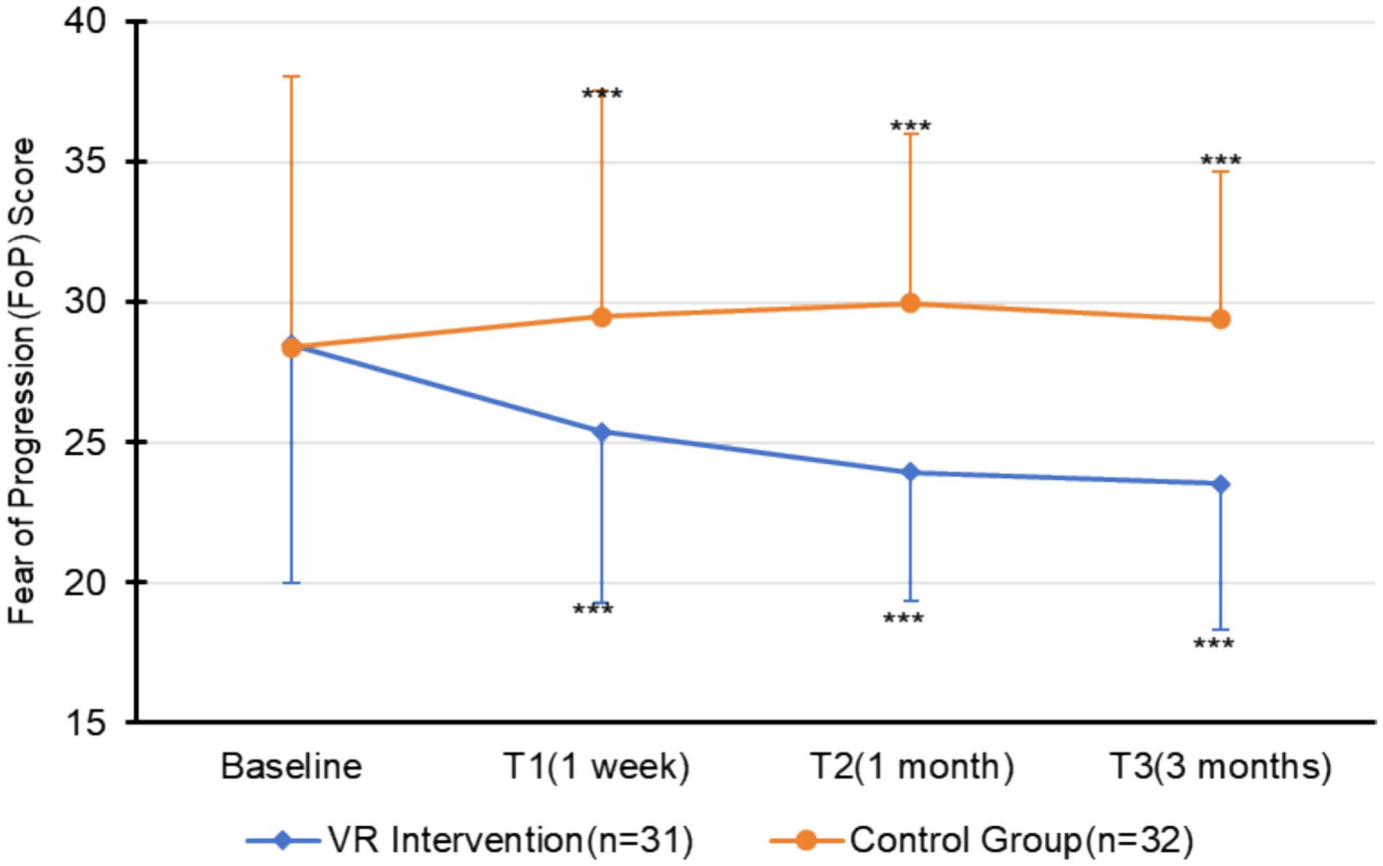
Temporal trends in fear of cancer recurrence scores across follow-up points. Error bars = standard deviations; ***p < 0.001. Time points: Baseline (pre-intervention), T1 (1 week), T2 (1 month), T3 (3 months).

A one-way ANCOVA was used to compare FCR scores between groups at 1 week (T1), 1 month (T2), and 3 months (T3), adjusting for baseline FCR, IU, and hope. Figure 4 visually depicts the temporal trend of FCR scores in the two groups. All models showed excellent fit (adjusted *R^2^* = 0.827–0.927), with Levene’s tests confirming variance homogeneity at T1 (*F* = 0.014, *p* = 0.907) and T2 (*F* = 0.503, *p* = 0.481); mild heterogeneity at T3 (*F* = 9.327, *p* = 0.003) did not affect validity due to ANCOVA’s robustness to minor violations. As shown in Table 2, after adjustment, the intervention group had significantly lower FCR scores than the control group at all time points.

**Table 2:** Main effects of VR intervention on fear of cancer recurrence at different time points.

Group	FCR (T1)	FCR (T2)	FCR (T3)
VR Intervention (*n* = 31)	25.39 ± 6.11	23.94 ± 4.59	23.52 ± 5.17
Control (*n* = 32)	29.50 ± 8.06	29.97 ± 6.05	29.38 ± 5.27
F (*df1, df2*)	66.75 (1, 61)	88.30 (1, 61)	93.55 (1, 61)
Partial *η^2^*	0.535	0.604	0.617
Model *Adj. R^2^*	0.927	0.827	0.84

Mean ± SD values represent the Fear of Progression (FoP-Q-SF) scores at each corresponding time point. ANCOVA models were adjusted for baseline FCR, illness uncertainty, and hope level; df₁ = numerator degrees of freedom (group effect); df₂ = denominator degrees of freedom (error term). Partial η^2^ = effect size indicator; values≥0.5 denote large effect sizes. All *p*-values for group main effects are <0.001.

The FCR scores of the intervention group decreased steadily from 28.48 at the baseline to 23.52 at T3, representing a 17.4% relative reduction from baseline, and no rebound was observed. In contrast, the scores of the control group remained consistently within the range of 28 to 29 throughout the follow-up period. These findings confirm that the VR intervention significantly reduces FCR levels and sustains clinical benefits for 3 months. The large effect sizes (all partial *η^2^* > 0.5) highlight its clinical relevance for patients with gynecological cancer.

### 3.3 Analysis of the parallel mediating effect: temporal dynamic evolution of the underlying mechanisms

**Figure 5:**
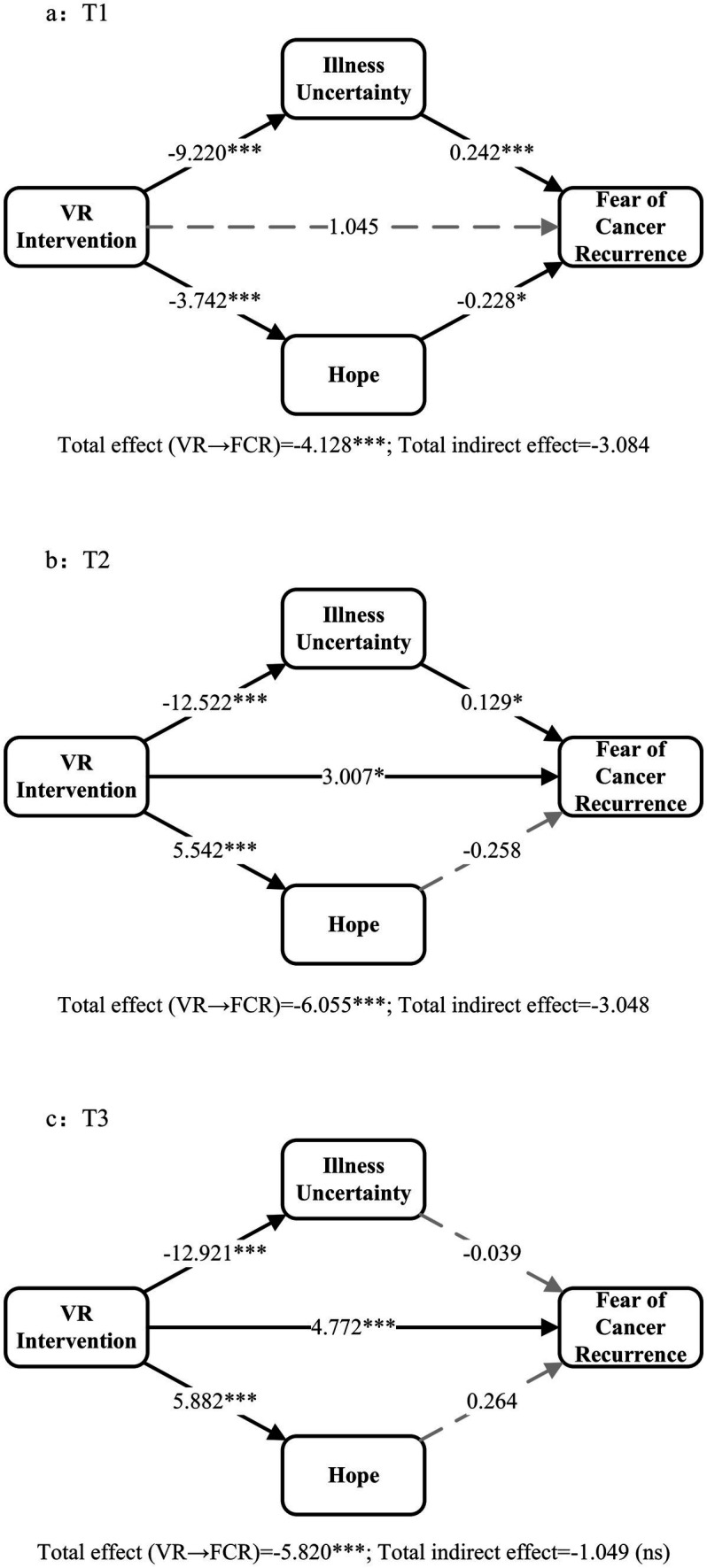
Time-dynamic parallel mediation pathways. *X* = Group (1 = VR intervention, 0 = Control); *M*_1_ = Illness uncertainty; *M*_2_ = Hope; Y = Fear of cancer recurrence (FCR). Bootstrap samples = 10,000. Solid lines = significant pathways (95% CI excludes 0); dashed lines = non-significant. All models adjusted for baseline covariates.

To examineTo examine how the VR intervention can reduce FCR, we used PROCESS Model 4 to examine the parallel mediation effects of IU (*M₁*) and Hope (*M₂*). The groups (VR intervention vs. control) were taken as the independent variable, while the FCR scores at 1 week (T1), 1 month (T2), and 3 months (T3) post-intervention were regarded as dependent variables. The baseline FCR, Hope, and IU scores were used as covariates. The bootstrap sampling (10,000 samples) combined with a 95% confidence interval was used to evaluate the indirect effect. All models showed excellent fit (adjusted *R^2^* = 0.858–0.956), supporting the reliability of the result. The temporal evolution of mediation pathways is illustrated in Figure 5. The detailed path analysis results, including the direct and indirect effects at each time point, are shown in Table 3.

**Table 3:** Parallel mediating effects of illness uncertainty and hope on fear of cancer recurrence.

Time	Paths	β	*SE*	*p*	95% Boot CI
T1	*X → M_1_*	−9.22	0.949	<0.001	–
	*X → M_2_*	3.742	0.542	<0.001	–
	*M_1_ → Y*	0.242	0.063	<0.001	–
	*M_2_ → Y*	−0.228	0.110	0.042	–
	Total effect	−4.128	0.505	<0.001	–
	Direct effect	−1.045	0.689	0.135	–
	*M_1_*	−2.231	0.549	–	[−3.319, −1.140]
	*M_2_*	−0.853	0.405	–	[−1.531, −0.061]
	Indirect effect	−3.084	0.604	–	[−4.182, −1.812]
T2	*X → M_1_*	−12.522	1.300	<0.001	–
	*X → M_2_*	5.542	0.479	<0.001	–
	*M_1_ → Y*	0.129	0.063	0.044	–
	*M_2_ → Y*	−0.258	0.171	0.137	–
	Total effect	−6.055	0.644	<0.001	–
	Direct effect	−3.007	1.291	0.023	–
	*M_1_*	−1.619	0.802	–	[−3.079, 0.079]
	*M_2_*	−1.428	0.983	–	[−3.253, 0.638]
	Indirect effect	−3.048	1.229	–	[−5.137, −0.335]
T3	*X → M_1_*	−12.921	1.091	<0.001	-
	*X → M_2_*	5.882	0.537	<0.001	-
	*M_1_ → Y*	−0.039	0.072	0.587	-
	*M_2_ → Y*	−0.264	0.145	0.074	-
	Total effect	−5.82	0.602	<0.001	-
	Direct effect	−4.772	1.366	0.001	-
	*M_1_*	0.505	0.987	–	[−1.464, 2.509]
	*M_2_*	−1.554	0.935	–	[−3.220, 0.449]
	Indirect effect	−1.049	1.340	–	[−3.512, 1.883]

X = Group (1 = VR intervention, 0 = Control); M₁ = Illness Uncertainty; M₂ = Hope; Y = Fear of Cancer Recurrence. Bootstrap samples = 10,000. 95% CI reported for indirect effects only. All coefficients adjusted for baseline FCR, illness uncertainty, and hope.

#### 3.3.1 M → Y paths

The *M* → *Y* paths exhibited distinct temporal dynamic attenuation. At T1, both mediation paths were significant: *M₁* → *Y* (IU → FCR) = 0.242 (*p* < 0.001) and *M₂* → *Y* (Hope→FCR) = −0.228 (*p* = 0.042). At T2, only *M₁* → *Y* remained significant (0.129, *p* = 0.044), while *M₂* → *Y* became non-significant (−0.258, *p* = 0.137). At T3, both paths were non-significant: *M₁* → *Y* = −0.039 (*p* = 0.587) and *M₂* → *Y* = −0.264 (*p* = 0.074).

#### 3.3.2 Indirect and direct effects

The total indirect effect showed a gradually decreasing trend over time: at T1, it accounted for 74.7% of the total effect, with both hope-mediated and IU-mediated pathways contributing significantly; at T2, this proportion dropped to 50.3%, mainly driven by the IU-mediated pathway; by T3, indirect effects were no longer significant, and the intervention’s impact was predominantly direct (82.0%).

The dual mediation mechanism exhibits a distinct temporal dynamic evolution pattern. In the early phase of intervention (T1), the VR intervention mitigates fear of relapse through two concurrent pathways: “enhancement of positive psychology” and “reduction of cognitive uncertainty.” During the middle phase (T2), the cognitive adjustment pathway remains dominant, whereas the mediating effect of positive psychological enhancement substantially diminishes. In the long-term phase (T3), the mediation effect dissipates, indicating that the sustained impact of the intervention is maintained primarily through its direct effect.

### 3.4 Chained mediation analysis: the transmission pattern of cognition-positive psychology

The PROCESS Model 6 was employed to test the chain mediation model (“*X* → *M₁* → *M₂* → *Y*”). The strength of the core mediation path “IU → Hope” (*M₁* → *M₂*) decreased significantly as time elapsed: it was significant at T1 (*β* = −0.237, *p* = 0.001), which supported a sequential mechanism from reducing IU to enhancing hope and ultimately alleviating FCR. However, it became non - significant at T2 and T3 (*p* > 0.05), resulting in a non - sustained chained mediation effect. As time went on, hope presumably developed into a stable trait, weakening its dynamic association with IU. Meanwhile, long-term benefits were mainly maintained through the direct effects of the intervention.

## 4 Discussion

This randomized controlled trial demonstrates that a chronotype-based timed VR intervention significantly reduces fear of cancer recurrence among gynecological cancer patients, with effects sustained up to 3 months post-intervention. Beyond establishing efficacy, the study reveals how psychological mechanisms evolve over time: illness uncertainty and hope serve as parallel mediators in the early phase, whereas the direct effect becomes increasingly dominant during follow-up. This temporal dynamic pattern offers new insights into the maintenance of intervention benefits and informs the design of booster strategies.

Fear of cancer recurrence among gynecological cancer patients represents a distinct psychological burden shaped by the intersection of disease-specific factors and gender-related concerns. Cervical and ovarian cancers not only threaten survival but also compromise reproductive function, disrupt hormonal balance, and alter body image. These treatment-related consequences intertwine with deeper psychosocial anxieties, including concerns about feminine identity and family role functioning (Dokras, 2024). Consequently, FCR in this population extends beyond a simple stress response, manifesting as a persistent psychological state characterized by perceived loss of control over the future, heightened vigilance to bodily sensations, and maladaptive avoidance behaviors (Pizzo et al., 2024; Sharpe et al., 2023).

Post-surgical hormonal fluctuations may further contribute to diurnal emotional instability in this population. Conventional psychological interventions, while providing fundamental support, often fail to account for individual variations in emotional regulation capacity and cognitive processing across the day (Sun et al., 2022). Addressing this gap, the present study integrated circadian rhythm assessment into the intervention framework, creating a model that aligns physiological rhythms with technology-delivered psychological support. The VR intervention group showed significantly lower FCR scores compared with controls at both T1 (mean difference = 4.11) and T3 (mean difference = 5.86). These findings align with prior evidence that VR technology can alleviate psychological distress in cancer populations (Shin et al., 2023), extending this evidence to gynecological cancer care. The chronotype-based timing approach may enhance intervention acceptability, though this was not directly assessed. More importantly, integrating evidence-based therapeutic modules with immersive VR technology appears to support the long-term maintenance of treatment benefits (Liu et al., 2023; Zhou et al., 2022).

Regarding mediating mechanisms, this study extends existing research by revealing how dual pathways evolve over time. At T1 (1 week post-intervention), reductions in illness uncertainty and increases in hope operated as parallel mediators through which the VR intervention reduced FCR. This pattern aligns with the Common Sense Model of Self-Regulation, which proposes that cognitive appraisals and emotional responses jointly shape coping behaviors (Hagger and Orbell, 2022; Ivynian et al., 2024). Illness uncertainty, as a core cognitive factor, influences how patients interpret their disease and respond emotionally (Parker et al., 2013; Shen et al., 2024). The VR intervention likely addressed cognitive gaps through visualized disease education and treatment simulations, disrupting the cycle of uncertainty, worry, and fear. Hope, as a psychological resilience resource, became more accessible through personalized positive future scenarios within the VR environment, helping to counteract negative emotions (Xu and Li, 2025). However, these mediating effects diminished after 1 month (T2) and were no longer significant by 3 months (T3), with FCR reduction increasingly driven by direct intervention effects. This temporal shift may reflect a psychological adaptation process: early-phase benefits rely on external support to disrupt negative patterns, whereas later-phase maintenance depends on internalized self-regulation capabilities developed through the intervention. Hope may gradually function as an independent protective factor, consistent with long-term resilience patterns observed among cancer survivors (Cohen et al., 2021; Mathews et al., 2024). These findings suggest that intervention optimization should target both cognitive and emotional mechanisms initially, then shift toward consolidating emotional memories and maintaining treatment gains through brief VR scene reviews in later stages (Kieszkowska-Grudny et al., 2019; Otto et al., 2022).

Compared with traditional psychological interventions, this program offers advantages in clinical feasibility. Following surgery, gynecological cancer patients typically undergo multiple follow-up examinations, radiotherapy, and chemotherapy, resulting in fragmented schedules and reduced physical tolerance. Traditional face-to-face psychological treatments requiring fixed appointments and multiple clinic visits pose adherence challenges for this population (Schreiter et al., 2025). In contrast, the VR intervention is straightforward to administer and can be delivered either at home or in hospital settings. The 20-min session duration aligns with patients’ physical capacity, minimizing fatigue (Groninger et al., 2024; Shin et al., 2023). Circadian rhythm-based timing requires only a brief assessment of daily routines and does not demand additional medical resources. This low-burden, high-adaptability design addresses the common clinical challenge of patients wishing to engage in psychological support but struggling to maintain consistent participation, offering a practical solution for integrating psychological care into comprehensive cancer rehabilitation (Alvarado-Omenat et al., 2025; Holt et al., 2021).

### 4.1 Limitations and future directions

Several limitations are acknowledged when interpreting these findings. The three-month follow-up period, while sufficient to establish medium-term efficacy, may not capture long-term fluctuations in FCR that occur with clinical milestones, life stressors, or disease progression. Extended follow-up to 6–12 months is warranted to determine whether intervention benefits persist and whether the observed temporal pattern of mediating mechanisms remains stable or continues to evolve. Such data would also inform the optimal timing for booster interventions. The single-center design and sample size of 63 participants limit the generalizability of findings. Although the sample was adequate for detecting the primary intervention effects, gynecological cancer encompasses diverse subtypes (cervical, ovarian, endometrial) with distinct disease trajectories and psychosocial correlates of FCR. The current sample may not fully represent how these differences influence intervention responsiveness. Additionally, factors such as socioeconomic status, access to digital resources, and regional variations in cancer care delivery were not examined. These factors can affect engagement with VR-based interventions, and the single-center setting likely fostered relative homogeneity in care processes and patient characteristics. Future multi-center studies with larger, more heterogeneous samples are needed to establish external validity across diverse clinical settings and underserved populations.

Reliance on self-reported measures represents another limitation. Although validated scales were used to assess FCR, illness uncertainty, and hope, self-report data are susceptible to biases such as social desirability and response shift. Future studies could integrate objective physiological markers aligned with circadian rhythms, such as diurnal cortisol profiles, sleep architecture, or continuous HRV monitoring from the VR device. Such multi-modal assessment would strengthen mechanistic insights by linking psychological outcomes to biological changes. Additionally, patient experiences with VR adherence were not systematically explored. Qualitative investigation of barriers related to device comfort, session timing, or content relevance would inform strategies to optimize real-world implementation.

Future research should address these limitations through multi-center designs with larger, more heterogeneous samples to examine differential responsiveness across tumor subtypes, age groups, and care settings. Extending follow-up to 6–12 months would confirm intervention durability, a key consideration for integration into long-term cancer survivorship care. A hybrid approach combining quantitative outcomes, patient-reported experience measures, and circadian-aligned physiological markers would enhance understanding of both effectiveness and underlying mechanisms. Finally, optimizing digital features such as remote adherence monitoring, adaptive content based on patient feedback, and telehealth support could enhance scalability and clinical utility across diverse care settings.

### 4.2 Clinical and practical implications

Healthcare policies can enhance cancer care quality by embedding digital mental health tools, such as VR-based psychosocial support, into routine oncology services. Given the demonstrated feasibility of nurse-led delivery and alignment with irregular post-treatment schedules, policy support should prioritize targeted resource allocation. This includes funding for specialized training programs to equip oncology nurses with skills in chronotype assessment and VR device operation, as well as subsidies for healthcare organizations to procure user-friendly, biofeedback-integrated VR devices. Clinical practice guidelines for cancer psychosocial care could incorporate evidence-based, biological rhythm-aligned personalized strategies. Moving beyond one-size-fits-all approaches, guidelines should integrate recommendations for adaptive intervention timing and dynamic mechanism monitoring to ensure care responds to individual patient trajectories. Policy can also foster cross-disciplinary synergy to refine and scale such innovations. Partnerships between academic institutions, technology developers, and clinical care teams can standardize intervention content, validate cultural adaptability, and streamline data sharing. Policies addressing health equity should extend these resources to under-resourced settings, where access to specialized psychological care is often limited. Finally, policy frameworks might include provisions for long-term outcome monitoring to systematically track sustained benefits and inform ongoing refinements to funding and implementation strategies.

## 5 Conclusion

This study demonstrates that a chronotype-based timed VR intervention significantly reduces fear of cancer recurrence among gynecological cancer patients through dual pathways: reducing illness uncertainty and enhancing hope. Intervention effects are sustained up to 3 months post-intervention, with the direct effect becoming increasingly dominant over time. This approach integrates immersive VR technology with individual chronotype assessment, providing a feasible approach for oncological psychological care that can be incorporated into routine clinical workflows.

## Data Availability

The raw data supporting the conclusions of this article will be made available by the authors, without undue reservation.
