# Supplementary material for: Exploring the temporal dynamics of circadian rhythm-aligned VR intervention in mitigating fear of cancer recurrence: mediating roles of illness uncertainty and hope in gynecological cancer patients
Source: Front Psychol. 2026 Apr 13;17:1797203. doi: 10.3389/fpsyg.2026.1797203 (PMC13111264; doi:10.3389/fpsyg.2026.1797203)
Supplement: Supplementary file 1 [file Table_1.docx]

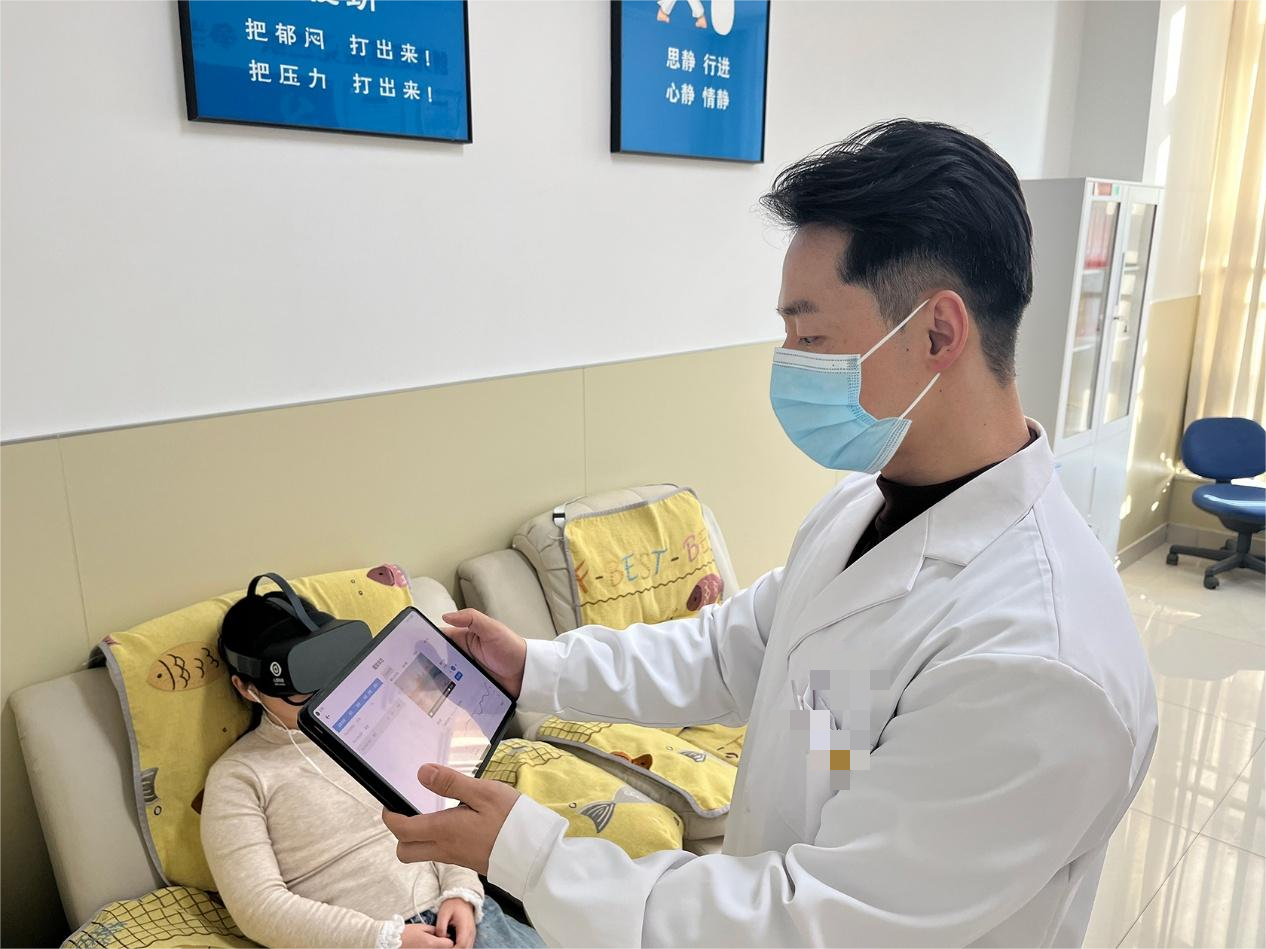
Supplementary 1: Chronotype-based timed VR intervention implementation: Structure and Content

Note: This figure shows a gynecological cancer patient using the VR device under clinical practitioner guidance, with real-time biofeedback monitoring via a tablet—demonstrating the intervention's clinical feasibility and operability. **The clinical practitioner monitored patient responses and biofeedback data via tablet throughout the session.

Supplementary 2 Personalized VR Psychological Intervention: Structure and Content

| **Week & Focus** | **Core Module** | **VR Activities & Environment** | **Session Structure** | **Therapist Responsibilities** | **Fidelity Measures** |
| --- | --- | --- | --- | --- | --- |
| Week 1  Cognitive Adaptation | Module 1: Cognitive Restructuring  (Self-Regulation Theory; CBT) | Activity: Psychoeducation on illness; identifying/challenging automatic negative thoughts about recurrence.  Environment: Calm, authoritative "virtual clinic" with clear visual medical information. | • 3-min introduction: Device setup & orientation  • 14-min core: Interactive cognitive exercises  • 3-min summary: Key point reinforcement | • Pre-session: Device calibration, patient comfort check  • During: Monitor engagement, provide encouragement  • Post-session: Debrief, answer questions | • Weekly supervision with PI  • 10% random session observations  • Fidelity checklist completion |
|  |  |  |  |  |  |
| Week 2  Emotion Regulation | Module 2: Emotion Regulation Training  (Mindfulness-Based Stress Reduction) | Activity: Mindfulness breathing, body scan; guided acceptance of anxiety.  Environment: Immersive natural landscapes (forest, beach, garden) to enhance sensory immersion and relaxation. | • 3-min introduction: Breathing preparation  • 14-min core: Guided mindfulness practice  • 3-min summary: Experience sharing | • Pre-session: HRV baseline measurement  • During: Real-time HRV monitoring, adjust pacing  • Post-session: Relaxation level assessment | • HRV data logging  • Session recording (10% sampled)  • Patient comfort rating |
|  |  |  |  |  |  |
| Weeks 3-4  Integration & Outlook | Module 3: Positive Future Visualization  (Hope Theory) | Activity: "Best possible self" imagery; constructing value-congruent future life scenes post-recovery.  Environment: Customizable "future life" scenes (family, work, leisure), allowing for personalization. | • 3-min introduction: Goal reflection  • 14-min core: Future scene immersion  • 3-min summary: Action planning | • Pre-session: Review T1/T2 assessment results  • During: Guide narrative engagement  • Post-session: Reinforce hope-related insights | • T1/T2 assessment integration check  • Personalization log  • Hope scale correlation |
|  |  |  |  |  |  |
